# Supplementary material for: The interactome of intact mitochondria by cross-linking mass spectrometry provides evidence for coexisting respiratory supercomplexes
Source: Mol Cell Proteomics. 2017 Dec 8;17(2):216–32. doi: 10.1074/mcp.RA117.000470 (PMC5795388; doi:10.1074/mcp.RA117.000470)
Supplement: Supplemental Data [file supp_RA117.000470_133922_0_supp_23978_fzffwf.zip › spectra_annotation/mito_DR_spectra_annotation/291-1-8-1-3-1.pdf]

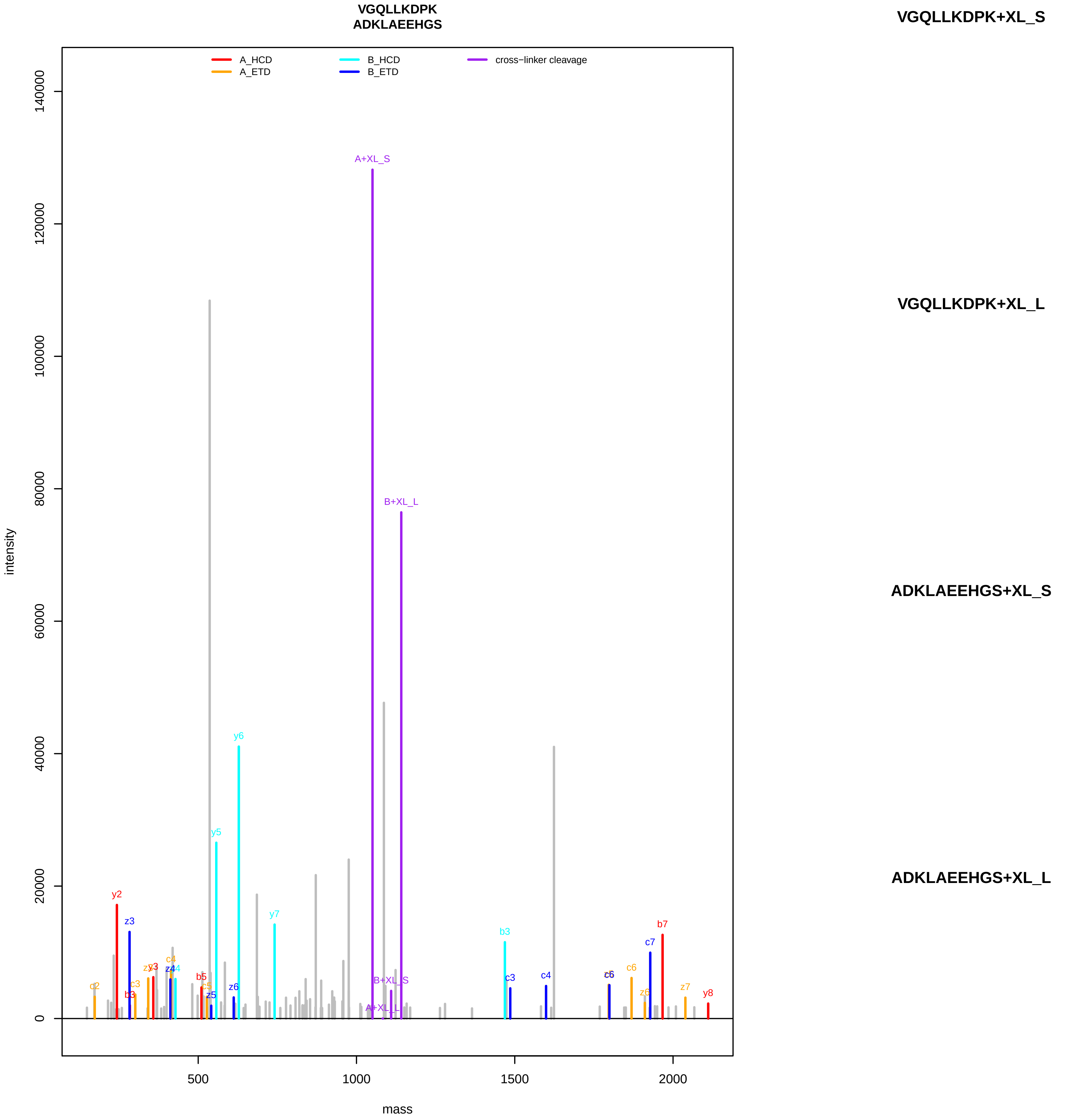
VGQLLKDPK
ADKLAEEHGS
A_HCD
A_ETD
B_HCD
B_ETD
cross−linker cleavage
intensity
mass
0
20000
40000
60000
80000
100000
120000
140000
500
1000
1500
2000
A+XL_S
B+XL_L
B+XL_S
A+XL_L
y2
z3
b3
c2
c3
z3
y3
c4
z4
y4
b5
c5
z5
z6
y5
y6
y7
b3
c3
c4
c6
c6
z6
c7
b7
z7
y8
VGQLLKDPK+XL_S
VGQLLKDPK+XL_L
ADKLAEEHGS+XL_S
ADKLAEEHGS+XL_L
